# User Experiences Among Patients and Health Care Professionals Who Participated in a Randomized Controlled Trial of E-nergEYEze, a Vision-Specific eHealth Intervention to Reduce Fatigue in Adults With Visual Impairment: Mixed Methods Study

**DOI:** 10.2196/53080

**Published:** 2025-08-07

**Authors:** Manon HJ Veldman, Hilde PA van der Aa, Hans Knoop, Christina Bode, Ger HMB van Rens, Ruth MA van Nispen

**Affiliations:** 1 Amsterdam UMC location Vrije Universiteit Amsterdam Ophthalmology Amsterdam The Netherlands; 2 Quality of Care, Mental Health Amsterdam Public Health Amsterdam The Netherlands; 3 Amsterdam UMC, location University of Amsterdam Departments of Medical Psychology Amsterdam Public Health research institute Amsterdam The Netherlands; 4 Department of Psychology Health and Technology University of Twente Enschede The Netherlands

**Keywords:** visual impairment, fatigue, eHealth, internet-based on cognitive behavioral therapy, iCBT, user experiences

## Abstract

**Background:**

Fatigue is a common symptom occurring in individuals with visual impairment (VI). Feeling fatigued has a strong impact on an individual’s well-being, with profound consequences. Cognitive and emotional functioning, social roles, and participation are negatively affected in severely fatigued individuals with VI. Therefore, we developed E-nergEYEze, a blended vision-specific eHealth intervention based on cognitive behavioral therapy and self-management to reduce fatigue severity in adults with VI.

**Objective:**

We aimed to report the experience of patients and professionals with E-nergEYEze. To complement cost-effectiveness outcomes, the user experiences from both perspectives were considered relevant for a better understanding of the intervention uptake.

**Methods:**

E-nergEYEze was studied in a randomized controlled trial. User experiences of participants with VI and severe fatigue (51/98, 52%; median age 58.0, IQR 53.0-65.0 years; female participants: 32/51, 63%), who were randomized to the intervention group, and professionals (n=11), who provided blended support, were evaluated. The Dutch Mental Health Care Thermometer questionnaire and a therapist evaluation were used and analyzed using mixed methods. A focus group meeting with social workers (4/7, 57%), a computer trainer (1/7, 14%), and psychologists (2/7, 29%) was held for more in-depth information. The eHealth platform provided data on user engagement from both perspectives.

**Results:**

E-nergEYEze was completed by 63% (32/51) of patients for more than 80% of the module steps. Overall, results on user engagement showed that a median 89% (IQR 45%-100%) of all assigned module steps were completed, with all modules being completed by at least 50% (37/51) of the patients. Completion of the intervention was related to the presence of digital proficiency; having the appropriate expectations; content that matches personal preferences and life context; and the absence of impeding personal circumstances, mental health issues, or other concurrent rehabilitation programs. The intervention was given a median grade of 7.0 out of 10.0 (IQR 6.0-8.0), and 87% (39/45) of the patients reported that they would recommend E-nergEYEze to others. However, improvements in the frequency and quality of guidance were considered highly relevant. Professionals reported that E-nergEYEze required patients’ self-efficacy, motivation, and digital skills; therefore, preselection was seen as essential. Professionals’ affinity with eHealth was considered important to provide appropriate remote support.

**Conclusions:**

eHealth provides treatment opportunities for individuals with VI for which guidance is considered highly relevant. During participation in E-nergEYEze, patients were engaged, internalized personally relevant topics, and made use of the benefits of eHealth. More attention to the suitability of patients and training of professionals for providing remote support is considered essential. These user experiences underlined the potential of E-nergEYEze to reduce fatigue severity in adults with VI and provided valuable insights to learn from and optimize E-nergEYEze.

**Trial Registration:**

International Clinical Trials Registry Platform (ICTRP) NL7764; https://tinyurl.com/32b3xt74

## Introduction

### Background

Fatigue is known to be an everyday problem for most individuals with visual impairment (VI) [1-4]. Consequences of fatigue on emotional and cognitive functioning, social roles, and participation in society have been reported to be problematic [5,6]. To address this difficulty, an intervention called E-nergEYEze was developed tailored to the unique aspects of individuals with vision loss who experience severe fatigue and investigated in a randomized controlled trial (RCT) [7]. E-nergEYEze is a blended eHealth intervention based on cognitive behavioral therapy (iCBT) and self-management. Professionals from low vision services offer online support and motivate patients to follow the intervention as intended. The goal is to teach patients how to cope with fatigue by focusing on understanding vision-specific fatigue, which makes them perceive it as more controllable and to reduce its consequences on daily life [5,8].

Considering the increasing demand for support in health care, eHealth offers a solution to reach a broader and larger audience. It can be offered independently of time and place, may stimulate patient empowerment, and requires less professional effort [9,10]. However, the challenge is to ensure that an intervention is consistent with patients’ needs and is easy for individuals with VI to use [11-13]. User experiences are of essential importance in all development and testing steps of iCBT to identify factors that drive participant behavior or affect outcomes and to gain a deeper understanding of participant experiences and needs [14]. Moss-Morris et al [15] conducted a pilot-RCT of a blended iCBT for individuals with multiple sclerosis (MS) in which qualitative feedback was reported on content and technical difficulties. Muijs et al [16] conducted a self-help iCBT intervention to prevent and reduce fatigue in adults with diabetes and reported the relevance of user experiences, stating that content and features need to be further personalized to preferences and needs. Finally, Babbage et al [17] studied a self-management app for fatigue in individuals with MS. Users reported challenges with the quantity and complexity of the text-based content. Results confirmed the relevance of qualitative feedback and the perceived value of fatigue-reducing iCBT tools.

The perspective of professionals is equally important to consider, especially because Dijksman et al [18] reported that 30% of psychologists did not believe that blended care had advantages in effectiveness and time savings compared to treatment as usual. However, overall, professionals were positive toward blended care, with significant differences in favor of active users versus nonusers. Because guidance skills, time, and input could influence outcomes [15,19], educational activities regarding eHealth, patient perceptions, and liability are considered necessary before using a new blended care intervention [18].

### Study Objectives

E-nergEYEze was designed in collaboration with all stakeholders (ie, patients, professionals, and researchers in low vision, fatigue, and eHealth). To maintain involvement in future iterations, this newly developed iCBT intervention was first pilot-tested in a usability and feasibility study by considering fidelity. Results showed a promising trend to reduce severity and impact of fatigue. The study methods were feasible, and the fidelity of the intervention protocol was suitable [20]. As the intervention was only studied partially to refine E-nergEYEze, we planned to further explore stakeholder experiences by offering the full intervention while focusing on its fatigue-reducing accessibility, content, and guidance. To complement the future cost-effectiveness outcomes, our aim was to evaluate the user experiences of the E-nergEYEze intervention from the perspective of patients and professionals.

## Methods

### The E-nergEYEze Protocol

This study was conducted according to the E-nergEYEze protocol [7].

### Study Design and Participants

Recruitment with information letters was performed by low vision service organizations in the Netherlands (Royal Dutch Visio and Bartiméus) and additionally through social media, patient associations, and 2 companies that provide low vision aids. Patients with VI and severe fatigue were included (N=98) in a single-blinded RCT and randomized to the intervention group (n=51, 52%) or control group (n=47, 48%). Patients were screened on the inclusion criteria having a mild VI (logarithm of the minimum angle of resolution visual acuity 0.30-0.40 or peripheral vision loss of <45°) or worse caused by an ophthalmic disorder; experiencing severe fatigue (a score on the Checklist Individual Strength questionnaire’s fatigue severity subscale of ≥35) [21]; understanding the Dutch language; having access to the internet; and being aged ≥18 years. Exclusion criteria were having severe cognitive impairment, as assessed with a 6-item screener (the validated 6-item Mini-Mental State Examination, with a cutoff score of >3) [22] and receiving treatment or having received treatment in the past 12 months from a medical specialist for a comorbid disease that could explain the presence of fatigue (eg, MS, cancer, or psychiatric disorder). A process evaluation was performed in patients who received the intervention (n=51, 52%) and professionals (n=11) of low vision service organizations who provided support. Both social workers (n=7) and computer trainers (n=4) kept time records, and the eHealth platform (e-platform) provided data on user engagement. A mixed methods design was used.

### Intervention

Patients were invited for a face-to-face session with a social worker and a computer trainer. Access was given to the e-platform Minddistrict [23] to follow an introduction module and 8 thematic modules with information and assignments on how to cope with fatigue based on cognitive behavioral therapy and self-management. The module themes and content are provided in Textbox 1.

**Module themes and content of the eHealth platform.**

**Introduction: Understanding vision-related fatigue**

It provides psychoeducation on vision impairment (VI) and its association with fatigue, including perpetuating factors of chronic fatigue.

**Module 1: Dealing with VI**

It focuses on the emotional impact of vision loss, processing feelings of grief, and coping strategies.

**Module 2: Formulating helpful fatigue-related beliefs**

It helps to understand the effect of beliefs on fatigue and fatigue maintaining behavior with exercises to formulate helping cognitions and shifting attention away from fatigue.

**Module 3: Graded activity program**

It focuses on balancing activities followed by a graded activity program.

**Module 4: Communication and social support**

It informs on the understanding of the role of verbal and nonverbal communication in the association between VI and fatigue with exercises aimed at fostering independence from others, facilitating adjustments in social relationships, and cultivating assertiveness.

**Module 5: Relaxation**

It helps to recognize stress and how to release tension by relaxation exercises.

**Module 6 (optional): Improving sleep**

It concentrates on understanding the importance of a fixed sleep-wake cycle, obtaining a personalized optimal sleep-wake cycle, and useful strategies to sleep or stay awake.

**Module 7 (optional): Work optimization**

It creates insight in balancing work-related stressors and energy sources and offers exercises to improve self-efficacy and possibilities for work resumption.

**Module 8: The future**

It focuses on achieving personal goals for the future, recognizing signs of relapse, and experiencing healthy levels of fatigue.

Patients were able to log in to their computer, tablet, or smartphone at their preferred place. Content was represented in module steps: the steps within a module that contained information or assignments. The e-platform logged how often, what content, and for how long patients were active over a period of 12 months. Social workers gave online feedback on assignments, and psychologists (n=2) were available for peer consultation.

### Outcome Measures

A survey was conducted using the Dutch Mental Health Care Thermometer (MHT) questionnaire [24] after 6 months by telephone to evaluate patients’ experiences. This questionnaire consisted of yes or no questions with regard to dimensions specified in the subscales: appreciation of information, appreciation of decision-making, and appreciation of professional and treatment results. Internal consistency of the subscales ranges between 0.70 and 0.86 [25]. Researchers (PhD candidates) from Amsterdam University Medical Centers conducted the assessments by telephone with registration into data-entry software called Castor [26]. There was no relationship established between researchers and patients before the study commencement. The e-platform provided data on user engagement.

Blended care is a relatively new way of providing guidance for professionals of low vision services in the Netherlands. The exploration of the experience of social workers and computer trainers with blended care and with guiding patients who follow the E-nergEYEze modules enabled a deeper understanding of insight into the professional perspective toward the digital aspect and content of the E-nergEYEze intervention within this target group. Therefore, social workers (n=7) completed the therapist evaluation for each patient. This questionnaire included questions on their experience with the intervention, online guidance, and compliance of patients to the intervention. Computer trainers (n=4) kept time records and evaluated the computer training. To complement the therapist evaluation outcomes, a focus group meeting was held with the involved social workers (n=4), computer trainer (n=1), and psychologists (n=2).

### Data Analysis

Careful consideration was given to information related to whether the intervention was completed. We decided to distinguish between patients who completed most of the module steps (≥80%) and patients who discontinued earlier (<80%). Quantitative data were analyzed using descriptive statistics, including baseline fatigue severity. Data were not normally distributed; therefore, results were reported with medians and IQR. The Mann-Whitney *U* test was performed to examine group differences in continuous variables and the chi-square test for nominal and ordinal variables. Qualitative data were analyzed by applying a thematic approach in MaxQDA2020 (VERBI Software) [27], a method to identify, analyze, and report patterns within data. To strengthen the reliability and validity of the analyses, MV (medical doctor and PhD candidate) and HvdA (senior researcher) separately analyzed answers to open questions of 3 evaluation forms to create thematic codes for either perspective, after which 2 code trees were created. All evaluations were then coded by MV, and data were analyzed by interpreting patterns within and between themes. Coding, analyses, and results were discussed in detail with HvdA.

Results from the professional perspective formed the content of the focus group meeting which was organized with 3 moderators (RvN, HvdA, and MV). Interaction among the professionals was encouraged with the leading questions “What goes well?” and “What can be improved and how?” This session was audio-recorded and transcribed verbatim by MV. Data were coded in vivo and analyzed thematically, using the preexisting code tree as a guide, and returned to the professionals for additional comments.

### Ethical Considerations

Ethics approval was obtained by the Medical Ethical Committee of the VU University Medical Center Amsterdam, the Netherlands (reference no. 2019/027, NL67802.029.18). The COREQ (Consolidated Criteria for Reporting Qualitative Research) checklist was applied in this manuscript [28]. Data was anonymized during data collection and analysis. Verbal consent was given by all professionals to record and transcribe the focus group meeting. All participants signed an informed consent form to participate in the study. Participants did not receive compensation for participation in the study.

## Results

Results reflect user experiences and user engagement of patients and professionals with the E-nergEYEze intervention.

### Participant Characteristics

Demographics and clinical characteristics of social workers and patients are presented in Tables 1 and 2, respectively. None of the variables were statistically different between patient subgroups. Clinically relevant differences were noticed in the subgroup who discontinued the intervention, including longer duration of the VI, less paid work, a higher percentage of participants living alone, and a history of psychiatric illness and comorbidities.

**Table 1:** Demographic and clinical characteristics of social workers (n=7) participating in the E-nergEYEze intervention.

Characteristics	Values
Age (y), median (IQR)	53.5 (49.0-62.0)
Sex (female), n (%)	6 (86)
Professional experience (y), median (IQR)	23.0 (13.0-31)
Working with individuals with VI^a^ (y), median (IQR)	12.0 (1.0-19)
Previous experience with eHealth (yes), n (%)	2 (29)

^a^VI: visual impairment.

**Table 2:** Demographic and clinical characteristics of patients with visual impairment participating in the E-nergEYEze intervention.

Characteristics		Total group (n=51)	>80% of module steps completed (n=32)	<80% of module steps completed (n=19)	*P* value	
Age (y), median (IQR)		58.0 (53.0-65.0)	57.5 (53.0-66.5)	59.0 (52.0-65.0)	.91^a^	
Duration of VI^b^ (y), median (IQR)		26.0 (14.0-47.0)	20.0 (10.8-41.3)	35.0 (19.0-51.0)	.14^a^	
Education (y), median (IQR)		12.0 (11.0-15.0)	11.5 (11.0-15.0)	12.0 (11.0-15.0)	.94^a^	
Fatigue severity score^c^, median (IQR)		42.0 (37.0-47.0)	43.5 (37.0-48.0)	40.0 (36.0-44.0)	.13^a^	
Sex (female), n (%)		32 (63)	21 (66)	11 (58)	.58^d^	
**Best-corrected eye^e^, n (%)**						.16^d^
	Blind	11 (22)	5 (16)	6 (32)		
	Severe VI	3 (6)	3 (9)	0 (0)		
	Moderate VI	26 (51)	15 (47)	11 (58)		
	Mild VI	11 (22)	9 (28)	2 (10)		
**Eyesight (subjective), n (%)**						.81^d^
	Blind	2 (4)	1 (3)	1 (5)		
	Very bad	21 (41)	12 (37)	9 (47)		
	Bad	20 (39)	13 (41)	7 (37)		
	Fair	8 (16)	6 (19)	2 (10)		
**Ophthalmic conditions^f^, n (%)**						—^g^
	Maculopathy	17 (33)	12 (37)	5 (26)		
	Retinopathy	17 (33)	9 (28)	8 (42)		
	Optic neuropathy	14 (27)	11 (34)	3 (16)		
	Anterior segment	4 (8)	2 (6)	2 (10)		
	Other^h^	9 (18)	4 (12)	5 (26)		
**Comorbidities, n (%)**						.13^d^
	None	28 (55)	21 (66)	7 (37)		
	1	13 (25)	6 (19)	7 (37)		
	≥2	10 (20)	5 (16)	5 (26)		
History of psychiatric illness, n (%)		12 (23)	6 (19)	6 (32)	.30^d^	
History of cancer, n (%)		6 (12)	3 (9)	3 (16)	.55^d^	
Hearing impairment^i^, n (%)		16 (31)	10 (31)	6 (32)	.64^d^	
**Work status, n (%)**						.44^d^
	Paid work	18 (35.3)	13 (41)	5 (26)		
	Volunteer work	14 (27.5)	7 (22)	7 (37)		
	No work	19 (37.3)	12 (37)	7 (37)		
Living alone, n (%)		14 (27.5)	6 (19)	8 (42)	.07^d^	
**Optional module, n (%)**						—
	“Improving sleep”	37 (72)	22 (69)	13 (68)		
	“Work optimization”	24 (47)	14 (44)	9 (47)		

^a^Denotes *P* values for the Mann-Whitney U test.

^b^VI: visual impairment.

^c^Fatigue severity score: severe fatigue ≥35 points.

^d^Denotes *P* values for the chi-square test.

^e^Best-corrected eye categories: blind: logarithm of the minimum angle of resolution (LogMAR) >1.30 or peripheral vision loss (<10°); severe vision impairment: LogMAR 1.30; moderate vision impairment: LogMAR 0.52 to 1.00 or peripheral vision loss (<30°); central visual field impairment or homonyme heminopia; mild vision impairment: LogMAR 0.30 to 0.40 or peripheral vision loss (<45°); and no visual impairment: LogMAR ≤0.22 or quadrantanopia.

^f^Patients can have one or more ophthalmic conditions at the same time.

^g^Not available.

^h^Other ophthalmic diseases: uveitis, infectious, scleritis, nystagmus, albinism, microphtalmia, evisceration, enucleation, trauma, iatrogenic, and acquired brain disease.

^i^Hearing impairment: subjective hearing being fair or bad.

### Experiences With the E-nergEYEze Intervention: Quantitative Results

#### eHealth Platform

Results on user engagement showed that median 89% (IQR 45-100) of all assigned module steps were completed (Table 3). In total, 32 (63%) patients completed most module steps (Tables 2 and 3) and graded the intervention median 8.0 (IQR 7.0-8.0). Modules were completed by at least 50% (37/51) of the participants and in both subgroups 50% (26/51) of the patients were active on the e-platform after completing the assigned modules. The number of patients that used the e-platform via the web browser and app was equally distributed. We noticed that the adherence to complete modules dropped below 50% after module 2 or during module 3 in the subgroup that discontinued the intervention, with little use of diaries (Tables 4 and 5). The diary on sleep-wake rhythm was used considerably more often compared to other diaries (Table 5). In addition, patients sent median 7.5 (IQR 4-12) messages and social workers sent median 12 (IQR 11-15) messages to their patients.

**Table 3:** User engagement data from patients who followed the E-nergEYEze intervention within the eHealth platform (n=51)^a^.

User engagement data	Total group (n=51), median (IQR)	>80% of module steps completed (n=32), median (IQR)	<80% of module steps completed (n=19), median (IQR)
Total number of module steps completed (%)	88.9 (44.7-100.0)	—^b^	—
Total time to complete the intervention (wk)	17.0 (11.0-24.0)	18.0 (13.3-25.0)	13.0 (6.0-24.0)
Time per module (wk)	2.8 (1.7-4.3)	2.3 (1.6-3.5)	4 (1.8-7.5)
Total time there was log-in activity (wk)	25.0 (18.0-38.0)	26.0 (19.8-46.0)	23.0 (14.0-33.0)
Total number of log-in times, n	35.0 (23.0-58.0)	49.5 (35.0-87.5)	22.0 (12.0-30.0)
Web browser use	16.0 (7.0-26.0)	20.0 (10.3-35.0)	9.0 (2.0-16.0)
Phone or tablet app use	15.0 (0.0-39.0)	22.5 (0.5-64.3)	6.0 (0.0-26.0)

^a^Three patients dropped out before registration on the eHealth platform.

^b^Not available.

**Table 4:** Modules use data from patients who followed the E-nergEYEze intervention within the eHealth platform (n=51)^a^.

Modules used	Total group (n=51), n (%)		>80% of module steps completed (n=32), n (%)			<80% of module steps completed (n=19), n (%)	
	Opened at least once	Completed 100%	Opened at least once	Completed 100%	Opened at least once		Completed 100%
Introduction: fatigue	48 (94)	44 (86)	32 (100)	30 (94)	16 (84)		14 (74)
Module 1: dealing with VI^b^	48 (94)	48 (94)	32 (100)	32 (100)	16 (84)		16 (84)
Module 2: negative thoughts	45 (88)	43 (84)	32 (100)	32 (100)	13 (68)		11 (58)
Module 3: graded activity	43 (84)	38 (74)	32 (100)	32 (100)	11 (58)		8 (42)
Module 4: social contact	37 (72)	33 (65)	32 (100)	30 (94)	5 (26)		3 (16)
Module 5: relaxation	38 (75)	32 (63)	32 (100)	31 (97)	6 (32)		1 (5)
Module 6 (optional): sleep	27^c^ (73)	22^c^ (60)	24 (100)	22 (92)	3 (24)		1 (8)
Module 7 (optional): work	16^d^ (67)	14^d^ (58)	15 (100)	14 (93)	1 (11)		0 (0)
Module 8: future	30 (59)	25 (50)	29 (91)	25 (81)	1 (5)		0 (0)

^a^Three patients dropped out before registration on the eHealth platform.

^b^VI: visual impairment.

^c^Module 6 was chosen by 37 patients.

^d^Module 7 was chosen by 24 patients.

**Table 5:** Diary use data from patients who followed the E-nergEYEze intervention within the eHealth platform (n=51)^a^.

Diaries	Total group (n=51)			>80% of module steps completed (n=32)			<80% of module steps completed (n=19)	
	Used at least once, n (%)	Number of times used, median (IQR)	Used at least once, n (%)		Number of times used, median (IQR)	Used at least once, n (%)		Number of times used, median (IQR)
Diary use	37 (72)	21.0 (13.0-42.0)	28 (87)		27.0 (20.0-54.0)	7 (37)		10.0 (4.0-18.0)
“My reaction”	35 (69)	5.0 (2.0-8.0)	26 (81)		6.5 (2.8-8.0)	7 (37)		4.0 (2.0-8.0)
“My helpful thoughts”	32 (63)	2.5 (1.0-5.8)	27 (84)		4.0 (2.0-6.0)	5 (26)		1.0 (1.0-2.5)
“My activity distribution”	31 (61)	4 (2.0-10.0)	27 (84)		5.0 (2.0-11.0)	4 (21)		1.5 (1.0-8.0)
“My activity build-up”	23 (46)	3.5 (1.0-11.0)	20 (62)		3.5 (1.3-11.0)	3 (16)		2.0 (1.0-1.0)
“My mental activity build-up” (optional)	18 (35)	2.0 (1.0-4.3)	16 (50)		2.0 (1.0-4.8)	2 (10)		1.0 (1.0-1.0)
“My social activity build-up” (optional)	17 (33)	2.0 (1.0-2.5)	14 (44)		2.0 (1.0-3.0)	3 (16)		1.0 (1.0-1.0)
“My relaxation”	26 (51)	4.5 (2.0-7.3)	25 (78)		5.0 (2.0-7.5)	1 (5)		3.0 (3.0-3.0)
“My sleep-wake rhythm” (optional)	18 (35)	12.5 (2.8-14.0)	17 (53)		13.0 (2.5-14)	1 (5)		3.0 (3.0-3.0)

^a^Three patients dropped out before registration on the eHealth platform.

#### Dutch MHT Questionnaire

E-nergEYEze was given a median grade of 7.0 (IQR 6.0-8.0) and patients put effort into following the intervention correctly with a median score of 8.0 (IQR 5.0-8.0). In total, 65% (30/46) of patients responded with “yes” to the question “Did you feel the intervention was the right approach for your problems or complaints.” The same number of patients (30/46, 65%) responded positively on 2 or more progress questions: “Did the intervention give you more control over your problems or complaints?” “Have you made sufficient progress as a result of the intervention?” “Are you better able to do things that matter to you as a result of the intervention?” and “Does the treatment help you cope better with people and situations, which you previously had problems with?” Some patients also indicated that they did not receive sufficient information about the intervention (5/46, 11%) and they felt that they were not given enough information about the expected outcome (16/46, 35%). Overall, most patients (39/45, 87%) reported that they would recommend E-nergEYEze to others.

#### Therapist Evaluation

Social workers spent median 190 (IQR 95-281) minutes per patient on preparation, making appointments, face-to-face session, online feedback, optional midterm or end evaluations, and peer consultation. The face-to-face session took a median 60 (IQR 33-90) minutes and providing feedback took a median 10 (IQR 10-15) minutes, with a median 8 (IQR 5-10) times per participant. Most contact with patients was online (79%) and by telephone (14%), and a small amount was live 4%). Social workers rated the effort patients (31/51, 61%) put into following the intervention correctly at a median of 8.0 (IQR 6.0-9.0). The computer trainer spent a median 30 (IQR 15-30) minutes on computer training with e-platform instructions. Additional computer training (n=11) was provided to 8 (N=51, 16%) patients for a median 15 (IQR 10-45) minutes, by telephone (7/11, 64%), online (2/11, 18%), and live (2/11, 18%). In total, 87% (7/8) of patients who requested additional computer training continued the intervention. Professionals provided a median grade between 7.0 and 8.0 (IQR 5.0-8.0).

### Experiences With the E-nergEYEze Intervention: Qualitative Results

#### Overview

The main themes derived from the qualitative data of the Dutch MHT questionnaire from the patient’s perspective (n=46), the therapist evaluation by social workers (n=7), and the focus groups with professionals (n=7) are summarized in Textbox 2. There were themes mentioned by the 3 stakeholder groups (patients, social workers, and professionals), for example, “effect and experiences,” 2 stakeholder groups, for example, “expectation, motivation, and time” that was mentioned by patients and social workers, or themes reflected by one group. Textbox 2 shows the themes reflecting the qualitative data of patients and professionals.

**Main themes derived from the qualitative data of patients and professionals regarding the E-nergEYEze intervention.**

**Patients (n=46)**

- Expectation, motivation, and time
- Content
- Guidance
- Digital accessibility
- Effect and experiences

**Social workers (n=7)**

- Expectation, motivation, and time
- Mental well-being
- Intervention delivery
- Providing digital support
- Effect and experiences
- Implementation suggestions

**Focus group meeting with professionals (n=7)**

- Intervention delivery
- Providing digital support
- Effect and experiences
- Implementation suggestions

#### Expectation, Motivation, and Time

Patients and social workers mentioned topics related to the theme “expectation, motivation, and time.” Feedback was given by patients (n=5, 11%) on the expectation of the intervention content and digital aspects: they would have liked more information upfront regarding module themes, the amount and length of the modules, and the eHealth design regarding the use of an online application. Some patients experienced a threshold to start the intervention (n=2, 4%) or had difficulties continuing the modules (n=1, 2%). Others mentioned that they had invested much time and effort to follow the modules (n=3, 6%) or would have liked more time per module (n=4, 9%). In total, 3 (6%) patients were of the opinion that the time investment was too great and 2 (4%) patients experienced difficulties attending E-nergEYEze in addition to having a job and mentioned a reduction in energy because of the increased awareness of fatigue being present. Social workers mentioned positive factors in participants’ continuation: having the appropriate expectations, a positive attitude, and completing the modules in time.

#### Content

Patients reflected positively on the intervention’s educational content, of which an important experience was being able to extract what was personally relevant to apply in daily life.

Assignments about how to distribute activities during the day, formulate helpful thoughts, apply relaxation exercises, avoiding sleep during the day, and optimize their work situation were reflected positively:

I put more attention on modules that were applicable and meaningful to me, namely: helping thoughts and distributing activities. I try to apply what I learned from these two modules in my daily life and I benefit from it.

Male patient, 55 y

There were difficulties with the amount and level of assignments and keeping diaries (n=8). In addition, patients (n=8, 17%) experienced insufficient progress because the content was either familiar or did not meet personal needs with regard to age, education level, or lifestyle. However, opinions varied on this topic—the intervention should be made more recognizable to the younger, socially active, and nondepressed people, or to more highly educated people as well as a better fit for older and less educated people. If the VI was already accepted, modules 1 and 2 seemed less compatible.

#### Guidance and Intervention Delivery

Patients were positive about the digital guidance (n=6, 13%) but also desired more frequent and substantive guidance (n=16, 35%). Illustrative examples included the following:

Consultation with the social worker could be better, there was limited response. I would have liked more interaction and shorter links with the social worker. For me, telephone contact would have helped.

Male patient, 62 y

Guidance was minimal and limited in content. I only received responses about being on track, but I felt the need to discuss whether I was succeeding and what my experiences were.

Female patient, 60 y

The computer training was experienced as supportive (n=2, 4%). However, some patients suggested that guidance was not necessary (n=3, 6%) or preferred the intervention in a face-to-face setting (n=4, 9%). Social workers mentioned that patients who gave no, evasive, or short answers, had no depth in their answers or those who were in need of supportive or empowering online feedback, were more likely to discontinue. Regarding intervention delivery, they experienced challenges with goal setting, providing online feedback, and remote support, especially with regard to assessing patients’ needs for the correct intensity and depth of online contact. Computer trainers (n=4) evaluated that most training sessions went well.

#### Mental Well-Being

When impeding personal circumstances or mental health issues were at play, patients struggled with continuing the intervention, for example, a changing home situation or the presence of mental or physical complaints caused by another illness.

#### Digital Accessibility and Providing Digital Support

Patients reflected positively on the eHealth design (n=4, 9%), with the possibility to reread information at a later time (n=2, 4%), and the diary reminders (n=1, 2%). However, digital skills among patients varied. Some patients mentioned no problems with the digital aspect of the intervention (n=4, 9%) and provided positive feedback on the presence of read-aloud audio within modules (n=3, 6%). Others mentioned inconveniences such as small font size (n=4, 9%) and no ability to flip the contrast (n=1, 2%) and had difficulties with navigating (n=4, 9%) and providing answers on the web (n=5, 11%). There were also patients who indicated that they had insufficient digital skills (n=2, 4%). Digital accessibility was mentioned as a barrier to progress (n=4, 9%). One of the responses included the following:

I am handy with my smartphone, but I found the app inconvenient for answering questions because some buttons were not read out loud or had to be operated in an illogical way. This was a stumbling point for me when following E-nergEYEze.

Female patient, 51 y

Social workers wondered, with regard to providing digital support, to what extent in-depth feedback was expected, how this should be shaped, and how to deal with evasive online contact. This is illustrated by the following quote:

I continue to find it difficult to provide proper feedback because from what is written I find it difficult to get clear to what extent a participant has really mastered the material. You don’t really get to a ‘conversation’ even when you do have more questions.

In contrast, only a few social workers consulted available psychologists for help. A total of 3 (43%) social workers indicated that they felt more comfortable with face-to-face contact and were of the opinion that affinity with eHealth is key.

#### Effect and Experiences

Patients experienced a positive change because of the intervention (n=9, 20%), for example, they gained more insight and awareness of problems based on the various topics covered in the intervention with valuable and educational information and personally relevant modules provided positive change in dealing with fatigue in daily life. Examples illustrating these results include the following:

In the past I was concerned with my eyes, but now in a different way. I have learned to think more positively. I am very enthusiastic about the intervention and have benefited greatly from several components.

Female patient, 60 y

I liked the e-learning as a tool very much, because I could read things a second or third time and therefore the content was better retained (vs. if it was presented orally). I was surprised that Module 1 was providing so much insight about the impact of low vision.

Female patient, 56 y

To the question “What do you think needs to be improved to increase the intervention grade?” the following responses were given:

Content was good, but I needed more personal guidance and more practical help tailored to my needs.

Female patient, 34 y

That the social worker was more responsive to the answers a person gave by mirroring for insight. There is also even more to be gained if guidance would capitalize more on specific problems given in answers to questions.

Female patient, 67 y

The intervention has to be changed, it was too long and intense. I felt a barrier to get started and felt the training burdensome for someone with fatigue.

Female patient, 58 y

Social workers felt the intervention had added value for patients and was a meaningful way to reduce fatigue in adults with VI. E-nergEYEze compelled thought and reflection by requiring users to fill in answers, allowing them to reread information, and allowing them to work at self-set time and pace. Patients gained insight and were able to make changes, especially if digital skills were present. In the opinion of the professionals, patients absorbed information of the different modules well. This was reflected by the following quote:

I thought it was good that my patient, while she had fatigue complaints since childhood and had developed a certain lifestyle, was still motivated and able to make changes with the help of the modules; for example she had instructed her environment not to ask about fatigue anymore.

#### Implementation Suggestions

Professionals indicated that motivation, self-discipline, and digital skills are a requirement to follow E-nergEYEze. Educational level must match content level for proper understanding and intended progress with clear expectations about the online aspect, intensity, and outcome of the intervention. Negative predictors were noted if patients had an unclear help request or personal goal, difficulties with the online aspect, and challenges with the content and diary assignments. In addition, it was felt necessary to exclude patients for whom mental issues or other rehabilitation needs play a prominent role. Therefore, critical preselection is important. Suggestions for improvements were made regarding prescheduled time blocks for feedback and peer consultation. Guidance should be approachable in a low-threshold manner with midterm and final evaluation. Evaluation moments could be used for the evaluation and reformulation of personal goals. It was discussed that fatigue requires attention early in the rehabilitation process, that E-nergEYEze is a fairly complete training, and that it provides neutral communication of information and tools to reduce severe fatigue.

## Discussion

### Principal Findings

This study evaluated the user experiences of the E-nergEYEze intervention, an iCBT intervention to reduce severe fatigue [14], from the perspective of patients with VI and professionals who used E-nergEYEze [7]. These firsthand experiences showed that patients completed a median of almost 90% of the module steps, almost two-thirds of the participants completed most of the intervention, and some were active up until 12 months from baseline. More than half of the patients were active on the e-platform at least 1 week after individually completing the last module step. This does not suggest that every patient finished all module steps but confirms that patients were actively engaged in the intervention and the potential of repetition offered by eHealth was noticed and applied to achieve long-term progress. Continuing E-nergEYEze was related to the presence of digital proficiency; having the appropriate expectation; content that matches personal preferences and life context; and the absence of impeding personal circumstances, mental health issues or concurrent other rehabilitation programs. However, it is challenging to find or develop an e-platform that is all-encompassing for the accessibility needs of individuals with VI.

E-nerEYEze was perceived as valuable to patients, especially personally relevant topics that triggered a positive change in dealing with fatigue. iCBT seems to be equally effective as face-to-face cognitive behavioral therapy [10], though we noticed an adherence drop in the subgroup that discontinued after module 2 or during module 3. Module 3, on graded activity, proved to be especially important in the reduction of fatigue [29,30]; however, this module is also relatively long and intensive, which may have caused patients to drop out. Adherence could be increased by motivating patients, embedding a midterm evaluation, or considering changing the order of modules. Reevaluating the difficulty of the content with professionals in cognitive behavioral therapy could be useful as well, for example, by reconsidering content of module 3 on the essence of physical activity to increase physical health and reduce fatigue. The examples of physical activities could be reconsidered in consultation with patient representatives to increase the feasibility of this module for individuals with VI and to lower the barrier to starting physical activities. The length of a module affects concentration and fatigue symptoms; however, a module does not have to be completed all at once. Practical alternatives to deal with length and intensity of content suggested by Babbage et al [17] were already implemented in the E-nergEYEze intervention*,* such as the use of voice-overs, read-aloud audio, and videos. We chose a blended design for E-nergEYEze design in consideration of the benefits of eHealth [9,10] and the more favorable outcomes that have been reported for guided iCBT versus unguided iCBT [31-33]. Our results showed that guidance was essential for patients; we underestimated the influence of and need for frequent and substantive support. Social workers were instructed to follow, motivate, and confirm patients’ progress in feedback moments, without extensive conversations on the costs and benefits of eHealth; this was seen as a discrepancy with patients’ needs. Worm-Smeitink et al [34] suggested including more flexibility in the intensity of guidance, a suggestion that we echo with respect to remote support. Our social workers had no experience in providing online support, and some struggled with the remote nature of eHealth, especially because of the limited interaction. Online interaction calls on other skills; therefore, skill, time and input could have influenced the treatment effects [15,19]. Precisely because eHealth is a new mode of treatment in this target population, it is important to pay adequate attention to the training of professionals providing low vision services and arrange prescheduled guidance time and peer consultation moments. In accordance with Dijksman et al [18], our results indicated that professionals need more support in providing substantive remote support.

### Strengths and Limitations of This Study

The major strengths of this study are presented in Textbox 3.

**Strengths of this study.**

- User experiences of the E-nergEYEze intervention were studied in the intervention arm of a single-blinded randomized controlled design.
- Quantitative and qualitative data were combined for optimal representation of practice.
- Stakeholders contributed to the development and guidance of E-nergEYEze to secure a pragmatic design.
- Internal validity was ensured in compliance with the study protocol.
- User experiences were analyzed ahead of the cost-effectiveness results.

We considered it a strength that these user experiences were analyzed ahead of the cost-effectiveness results, which confirm an independent analysis and interpretation of data. E-nergEYEze was developed by various professionals and patients, based on evidence-based iCBT [35,36] and was pilot tested [20]. Patients gave positive feedback on improvements made based on the pilot study, reflecting the value of pilot testing before conducting an RCT. The content variety gave patients the possibility to personalize their treatment by applying coping strategies that appealed to them in daily life, as well as broadening their horizon outside their comfort zone. E-nergEYEze has been implemented in compliance with the study protocol [7], ensuring the internal validity of these results with respect to the intervention. However, the reliance of self-reported time records of professionals could limit actual or perceived validity. Another limitation that should be considered is the possibility of self-assessment bias, that is, the overestimation of one’s own performance. The mixed method design included qualitative analysis; therefore, discussing reliability is challenging, but we have striven to be consistent in the analysis. Internal validity was enhanced by triangulation of research methods and verification of qualitative analysis. The sample size of this study was relatively small, as we zoomed in on the user experiences of the intervention group and the professionals who were involved. This could limit the generalizability of the results. In addition, attrition bias could have influenced the results, as 5 patients did not complete the Dutch MHT questionnaire.

### Implications

Our results respond to the knowledge gap regarding user experiences with blended iCBT in adults with VI [37]. To determine whether a patient receiving low vision services is suitable for E-nergEYEze, the difference in patient characteristics between the subgroups that we investigated may be of clinical relevance. Patients who discontinued the intervention could have benefited from receiving more support, for which training of professionals is essential. Guided iCBT has increasingly been investigated, with varying guidance intensity [15,36,38,39], and it would be valuable to examine what frequency and type of guidance can best be offered in iCBT, as well as the amount of content and how to match the difficulty to the skills and opportunities of patients. Taking our results into consideration, individuals with VI who experience severe fatigue, who are digitally sufficient, and who are willing to receive remote treatment with the ability and motivation to work at self-set times are likely to benefit from the E-nergEYEze intervention. Individuals with impeding personal circumstances, mental health issues, or who are participating in other concurrent rehabilitation programs are less likely to benefit or more likely to discontinue the E-nergEYEze intervention.

### Conclusions

The potential of the E-nergEYEze intervention to reduce fatigue severity in individuals with VI was underlined by user experiences. These results provide information to learn from and optimize E-nergEYEze into a useful, available, and affordable tool to reduce severe fatigue in individuals with VI. This research offers future prospects for the development of blended eHealth interventions for other conditions in this target group with specific needs regarding accessibility of e-platforms and guidance with an affinity for providing digital care.

## Abbreviations

COREQ: Consolidated Criteria for Reporting Qualitative Research
iCBT: intervention based on cognitive behavioral therapy
MHT: Mental Health Care Thermometer
MS: multiple sclerosis
RCT: randomized controlled trial
VI: visual impairment

## Data Availability

The nonidentifiable data and data collected directly from patients during this study are available from the corresponding author upon reasonable request.
